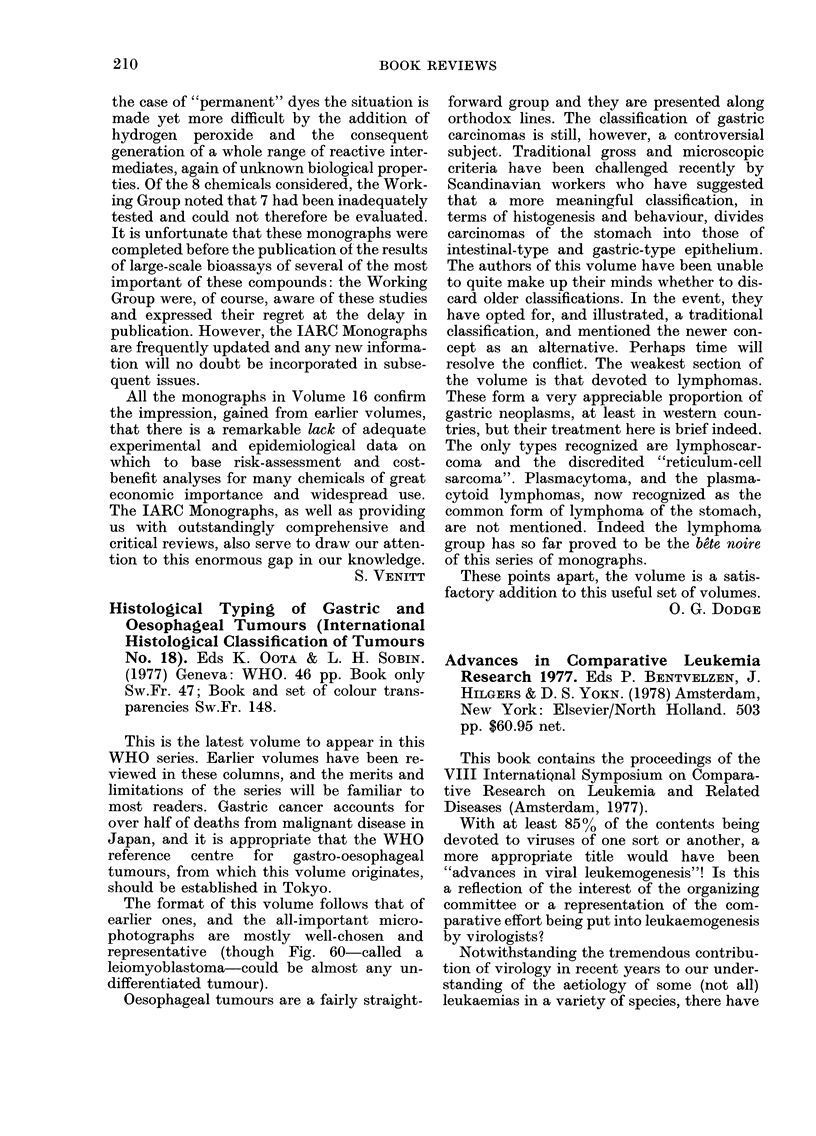# Histological Typing of Gastric and Oesophageal Tumours (International Histological Classification of Tumours No. 18)

**Published:** 1979-02

**Authors:** O. G. Dodge


**Histological Typing of Gastric and Oesophageal Tumours (International Histological Classification of Tumours No. 18).** Eds K. OOTA & L. H. SOBIN. (1977) Geneva: WHO. 46 pp. Book only Sw.Fr. 47; Book and set of colour transparencies Sw.Fr. 148.

This is the latest volume to appear in this WHO series. Earlier volumes have been reviewed in these columns, and the merits and limitations of the series will be familiar to most readers. Gastric cancer accounts for over half of deaths from malignant disease in Japan, and it is appropriate that the WHO reference centre for gastro-oesophageal tumours, from which this volume originates, should be established in Tokyo.

The format of this volume follows that of earlier ones, and the all-important microphotographs are mostly well-chosen and representative (though Fig. 60—called a leiomyoblastoma—could be almost any undifferentiated tumour).

Oesophageal tumours are a fairly straightforward group and they are presented along orthodox lines. The classification of gastric carcinomas is still, however, a controversial subject. Traditional gross and microscopic criteria have been challenged recently by Scandinavian workers who have suggested that a more meaningful classification, in terms of histogenesis and behaviour, divides carcinomas of the stomach into those of intestinal-type and gastric-type epithelium. The authors of this volume have been unable to quite make up their minds whether to discard older classifications. In the event, they have opted for, and illustrated, a traditional classification, and mentioned the newer concept as an alternative. Perhaps time will resolve the conflict. The weakest section of the volume is that devoted to lymphomas. These form a very appreciable proportion of gastric neoplasms, at least in western countries, but their treatment here is brief indeed. The only types recognized are lymphosarcoma and the discredited "reticulum-cell sarcoma". Plasmacytoma, and the plasmacytoid lymphomas, now recognized as the common form of lymphoma of the stomach, are not mentioned. Indeed the lymphoma group has so far proved to be the *bête noire* of this series of monographs.

These points apart, the volume is a satisfactory addition to this useful set of volumes.

O. G. DODGE